# A hybrid ICA-GRU approach for forecasting gold futures price

**DOI:** 10.3389/frai.2026.1934950

**Published:** 2026-09-10

**Authors:** Sirisha Charugulla, Shaiku Shahida Saheb

**Affiliations:** VIT-AP School of Business, VIT-AP University, Amaravati, India

**Keywords:** gated recurrent unit neural network, gold futures price prediction, hybrid deep learning models, Independent Component Analysis, time-series analysis

## Abstract

Accurate forecasting of gold futures prices is very important for investors and analysts due to the highly volatile and nonlinear nature of the commodity markets. This research work develops a hybrid ICA-GRU framework to gold futures price forecasting by developing a hybrid approach of ICA and GRU-based neural networks. The historical daily gold futures price data spanning from 2015 to 2026 were collected from Yahoo Finance. ICA transforms correlated financial variables into statistically independent components, generating informative latent feature representations that may better capture the underlying structure of financial time-series data for subsequent sequence learning. The GRU-based neural network was then applied to identify the nonlinear pattern in the data. The performance of the proposed ICA-GRU model was measured using RMSE, MAE, MASE and *R*^2^, precision, recall, confusion matrix, and significance testing. The experimental results demonstrated that the RMSE was 39.36, MAE was 26.35, MASE was 2.61 and *R*^2^ was 0.99, which was considerably better than that of the traditional GRU model. According to the results of the Wilcoxon Signed-Rank Test, the difference between model performances was statistically significant (*p* < 0.001). The obtained results suggest that the developed ICA-GRU model can be considered a promising tool for gold futures price forecasting.

## 1 Introduction

Gold holds a prominent place in the global financial system because of its dual role as both a commodity and a financial asset. It has long been regarded as a reliable investment and an effective hedge against inflation, currency fluctuations, and geopolitical uncertainty (Taneva-Angelova et al., 2025). During periods of financial instability, gold often serves as a safe-haven asset, attracting investors seeking to preserve wealth (Bunnag, 2024). In developing economies such as India, gold has additional cultural and economic significance, making accurate gold futures price forecasting imperative for investors, financial institutions, and policymakers. However, forecasting gold futures prices remains challenging because of the complex interactions among macroeconomic, financial, and geopolitical factors, including inflation, interest rates, exchange rates, stock market performance, global uncertainty, and central bank gold purchases (Zhou and Liang, 2025).

Several forecasting approaches have been proposed to address these challenges. Traditional econometric models provide theoretical interpretability but generally assume linear relationships and therefore have limited ability to capture the nonlinear dynamics of financial markets. Machine learning techniques have demonstrated predictive performance by modeling nonlinear relationships; however, they often require extensive feature engineering and may not adequately capture temporal dependencies (Liang et al., 2022). Recently, deep learning models, particularly recurrent neural networks such as Long Short-Term Memory (LSTM) and Gated Recurrent Unit (GRU), have achieved promising results in financial time-series forecasting by effectively learning sequential patterns directly from historical data (You et al., 2025). LSTM networks and similar recurrent architectures have demonstrated strong forecasting performance owing to their ability to learn temporal dependencies directly from data. Impartial LSTM models have been known to exhibit low forecasting error rates for both batch and real-time settings (Dalimunthe et al., 2025). Nevertheless, these models remain susceptible to noise, multicollinearity, overfitting, and the inherent volatility of financial markets (Foroutan and Lahmiri, 2024). To overcome these limitations, hybrid forecasting frameworks that combine statistical signal decomposition techniques with deep learning models have gained growing attention (Jabeur et al., 2024). Independent Component Analysis (ICA) has emerged as an effective preprocessing technique for transforming correlated financial variables into statistically independent components, thereby providing an alternative latent feature representation for subsequent deep learning models. This transformation may facilitate the extraction of informative patterns from financial time-series data, making ICA a suitable preprocessing approach for hybrid forecasting frameworks (Liang et al., 2022).

Despite significant advances in gold futures price forecasting, important research gaps remain. Existing studies have widely employed machine learning and deep learning models, including Support Vector Machines, Random Forests, Long Short-Term Memory (LSTM), Convolutional Neural Networks (CNNs), CNN-LSTM hybrids, and Transformer-based architectures. Although these approaches have demonstrated strong predictive capability, many remain sensitive to noise, multicollinearity, and market volatility. Furthermore, relatively few studies have investigated the integration of Independent Component Analysis (ICA) with Gated Recurrent Unit (GRU) networks for gold futures price forecasting. Consequently, the potential benefits of combining ICA-based signal decomposition with GRU-based temporal learning remain insufficiently explored.

The purpose of this study is to propose a hybrid Independent Component Analysis–Gated Recurrent Unit (ICA-GRU) model for gold futures price forecasting. The proposed ICA-GRU model integrates Independent Component Analysis with a stacked Gated Recurrent Unit architecture to transform correlated financial variables into statistically independent components and learn the temporal dependencies underlying gold futures price movements. Specifically, the study aims to: (i) transform correlated financial variables into statistically independent components using ICA to obtain an alternative feature representation, (ii) capture complex nonlinear relationships and temporal patterns using stacked GRU networks, and (iii) improve the accuracy, robustness, and reliability of gold futures price forecasting compared with existing forecasting approaches. By combining statistical signal decomposition with deep learning, the proposed ICA-GRU model provides a scalable and effective forecasting framework that can support informed decision-making by investors, financial analysts and policymakers.

The remainder of this paper is organized as follows: Section 2 reviews the existing literature on gold futures price forecasting. Section 3 presents the proposed methodology and the ICA-GRU model architecture. Section 4 discusses the experimental results and performance evaluation. Finally, Section 5 concludes the paper.

## 2 Literature review

Gold futures price prediction models that were considered in early research studies focused primarily on econometric and statistical methods because of their interpretability and capability to explain long-run relations. Typically, macroeconomic factors such as inflation rate, interest rate, and exchange rate are taken into account using these models. For example, distributed lag models and stochastic volatility models have been used for modeling long-memory properties and heavy-tailness of gold prices (Aqilah and Dini, 2024). Moreover, cointegration and multivariate models underline the dependence of gold prices, oil prices, and exchange rates (Beckmann and Czudaj, 2013; Beckmann et al., 2015). These approaches are theoretically strong but extremely limited by linear assumptions and parametric forms, which makes them ineffective for modeling nonlinear and very volatile markets. Thus, their prediction power is poor, especially during structural breaks. Even though these models have many advantages theoretically, their main drawback lies in their strong dependence on linear assumptions and parametric modeling, thus making them ineffective for nonlinear and volatile market conditions. Their prediction performance is severely affected by structural changes in regimes (Hashem Pesaran et al., 2006).

### 2.1 Traditional and econometric approaches

The previous studies employ econometric and statistical approaches to examine linear dependencies and long-term dynamics in gold prices. The distributed lag model has been employed to evaluate the impact of macroeconomic variables such as interest rates on national gold markets; reliable medium-term prediction yet low adaptability to nonlinear gold price changes (Aqilah and Dini, 2024). Similarly, stochastic and long memory volatility models such as ARFIMA-FIGARCH and α-stable stochastic differential equations have exhibited reliability in the modeling of gold prices’ heavy-tailed distributions and long-term dynamics (Coulibaly et al., 2024; Basira et al., 2024). While these methods are very reliable in terms of theoretical interpretations and useful for long-time analyses, they are less efficient under volatile and nonlinear market conditions due to their parametric nature.

### 2.2 Machine learning–based gold price prediction

In order to deal with the limitations of linear models, several works have resorted to the use of various types of machine learning algorithms such as SVM, RF, KNN and MLP. Studies have shown empirically that tree-based fusion models such as RF and XGBoost always perform better than conventional regression models because of the ability of the models to account for nonlinear relationships between gold prices and various cross-market variables (Tripurana et al., 2025; Jabeur et al., 2024). With the addition of explanatory means such as SHAP, the models become more interpretable by identifying the relative applicability of economic and financial predictors. However, most of the machine learning models do not handle sequential information explicitly or at all.

### 2.3 Deep learning and hybrid architectures

Recent research tends to employ deep learning approaches, specifically Long Short-Term Memory (LSTM) networks as well as other similar networks, owing to the ability to learn temporal dependency from the data directly. Impartial LSTM models have been known to exhibit low forecasting error rates for both batch and real-time settings (Dalimunthe et al., 2025). Nevertheless, hybrid deep learning models have consistently shown superior results. Research that involves CNNs combined with LSTM and Bi-LSTM models has proven increased forecasting accuracy through spatial feature extraction alongside temporal learning capacity (Amini and Kalantari, 2024). Hybrid LSTM-Transformer models with multilevel feature fusion enhance forecasting precision by capturing short-term and long-term dependencies of data through an attention mechanism (Zhao et al., 2025).

Moreover, decomposition models such as the VMD–GRU–CEEMDAN framework have exhibited great improvements (more than 30%) in forecasting accuracy via decomposition of gold price series into the trend and fluctuation parts (Zhang et al., 2025). Likewise, the LSTM Autoencoder approach exhibits great generalization ability and prevents overfitting in the high volatility of the gold market (Saini et al., 2025).

### 2.4 Incorporation of optimization, technical indicators, and big data

Other major research streams involve improving forecasting performance through technical indicators, optimization methods, and alternative data sets. Metaheuristic optimization methods include metaheuristic optimization algorithms like the artificial bee colony algorithm and bioinspired optimization has been applied for fine-tuning the hyperparameters of deep learning to achieve faster convergence and reduced forecasting errors (Huo et al., 2024; Pillai and Mary, 2025). Other technical indicators, including the Fibonacci retracement levels, have also been explored to increase the accuracy of the model when combined with machine learning and deep learning methods (Priambodo et al., 2025).

Other than numeric statistics, big data-driven methods involving text mining and sentiment analysis have emerged as another growing frontier. Models combining Google trends data, financial news, and deep learning methods are better at forecasting compared to econometric methods because they capture the sentiment and psychological aspects of markets (Poor et al., 2024). Sentiment-based real-time trading models have further proved the applicability of these methods by generating profitable trading results (Varela et al., 2025).

### 2.5 Safe-haven behavior and market uncertainty

Other researchers take the investigation of gold into the domain of hedging and safe-haven by means of multivariate stochastic volatility and uncertainty-driven approaches. In particular, it was found that the safe-haven properties of gold are time-varying and depend on the specific context, especially when it comes to global financial stress (Feder-Sempach et al., 2024). Such studies add to the economic explanation of gold price fluctuations; however, their major emphasis is put on the explanatory aspect rather than the predictive one. The study finds that investor attention by using DCC-RGARCH-X (Dynamic Conditional Correlation-Realized GARCH with Exogenous Variables) model, the study finds that investor attention, measured through Google searches for oil prices, significantly improves covariance forecasting between oil, gold and stock markets (Fiszeder et al., 2023).

Nevertheless, some gaps are obvious in the research on gold futures price prediction. First of all, there is no particular model that would excel in the majority of experiments (Varshini et al., 2024). Second, high-performing deep learning models are generally noninterpretable and sensitive to hyperparameters. Third, there are only a few studies where the signal decomposition, optimization, and deep recurrent architectures are considered together in one prediction model. Thus, there is a necessity for a robust and optimized hybrid prediction model that would capture nonlinear dynamics and reduce noise.

## 3 Research methodology

**Figure 1:**
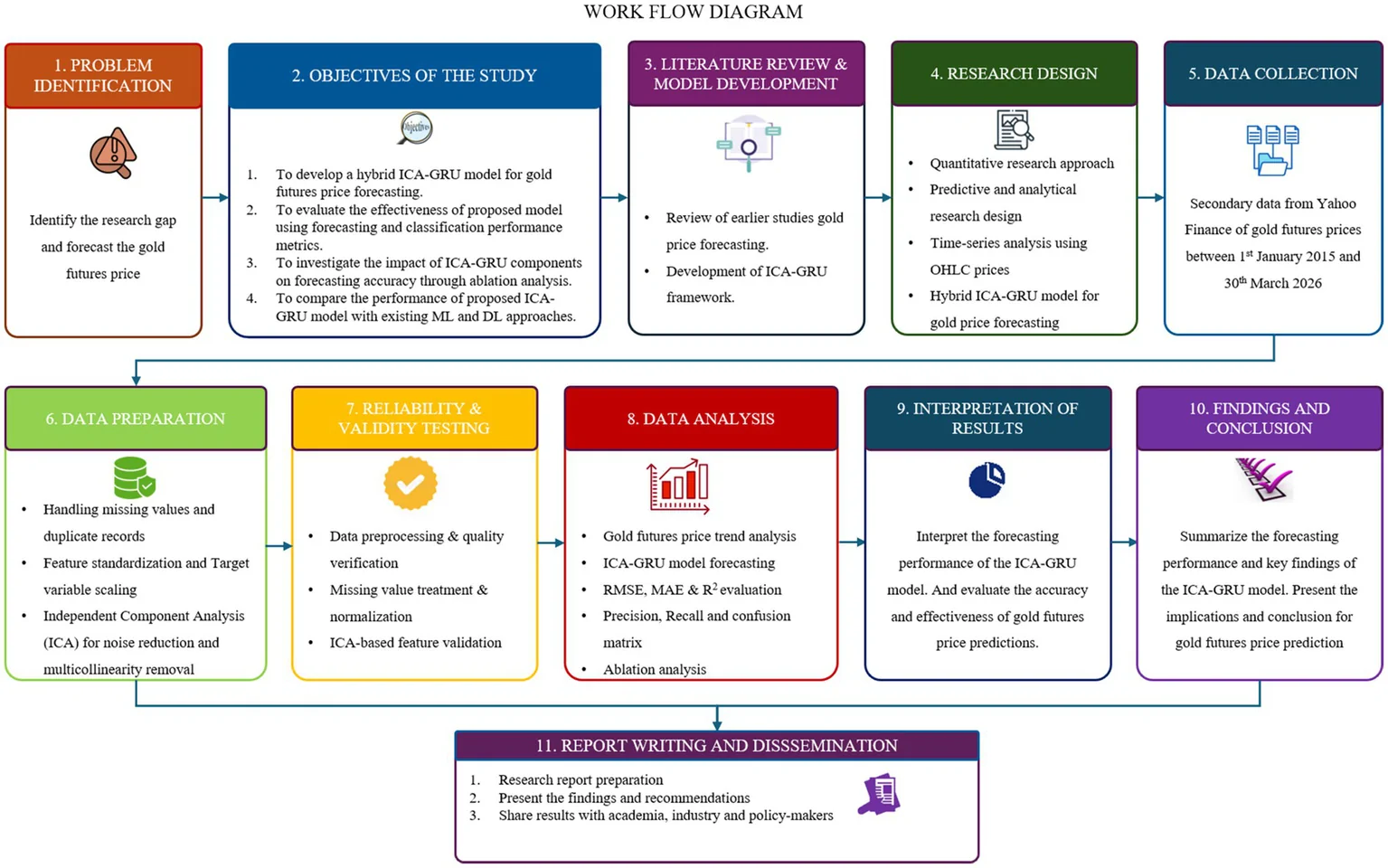
Gold futures price forecasting workflow diagram.

Figure 1 presents the overall research framework used to develop the proposed ICA-GRU model for gold futures price forecasting. The study begins by identifying the research problem and defining the research objectives. A comprehensive review of previous studies is conducted to identify research gaps and support the development of the proposed hybrid model.

Historical daily gold futures price data (2015–2026) are collected from Yahoo Finance and preprocessed by handling missing values, removing duplicate records, standardizing the data, and applying Independent Component Analysis (ICA) to improve data quality. The processed data are then used to train and evaluate the ICA-GRU model. Model performance is assessed using RMSE, MAE, *R*^2^, precision, recall, and a confusion matrix analysis.

### 3.1 Model development

The selection of the proposed ICA-GRU model is motivated by the complementary strengths of Independent Component Analysis and Gated Recurrent Unit networks for financial time-series forecasting. Financial time-series data, including gold futures prices exhibit strong correlations among market variables, resulting in redundant information that may adversely affect predictive performance (Liang et al., 2022). ICA transforms correlated variables into statistically independent components, thereby reducing feature redundancy and generating informative latent feature representations for subsequent learning (Hyvärinen and Oja, 2000). Unlike PCA, which maximizes variance through orthogonal transformations, ICA explicitly seeks statistical independence among extracted components (Jolliffe and Cadima, 2016). Although decomposition techniques like Variational Model Decomposition (VMD), Ensemble Empirical Mode Decomposition (EEMD) and Singular Spectrum Analysis (SSA) have demonstrated promising forecasting performance, they generally require iterative signal decomposition procedures and additional parameter tuning (Dragomiretskiy and Zosso, 2014; Zhang et al., 2025). In contrast, ICA directly transforms multivariate financial variables with comparatively lower computational complexity. The extracted independent components are subsequently processed by the GRU network, which efficiently captures nonlinear temporal dependencies through its gated architecture while requiring fewer trainable parameters than LSTM networks. Therefore, integrating ICA with GRU provides a computationally efficient hybrid framework capable of learning temporal dynamics from statistically independent feature representations, thereby improving forecasting performance for gold futures prices.

**Figure 2:**
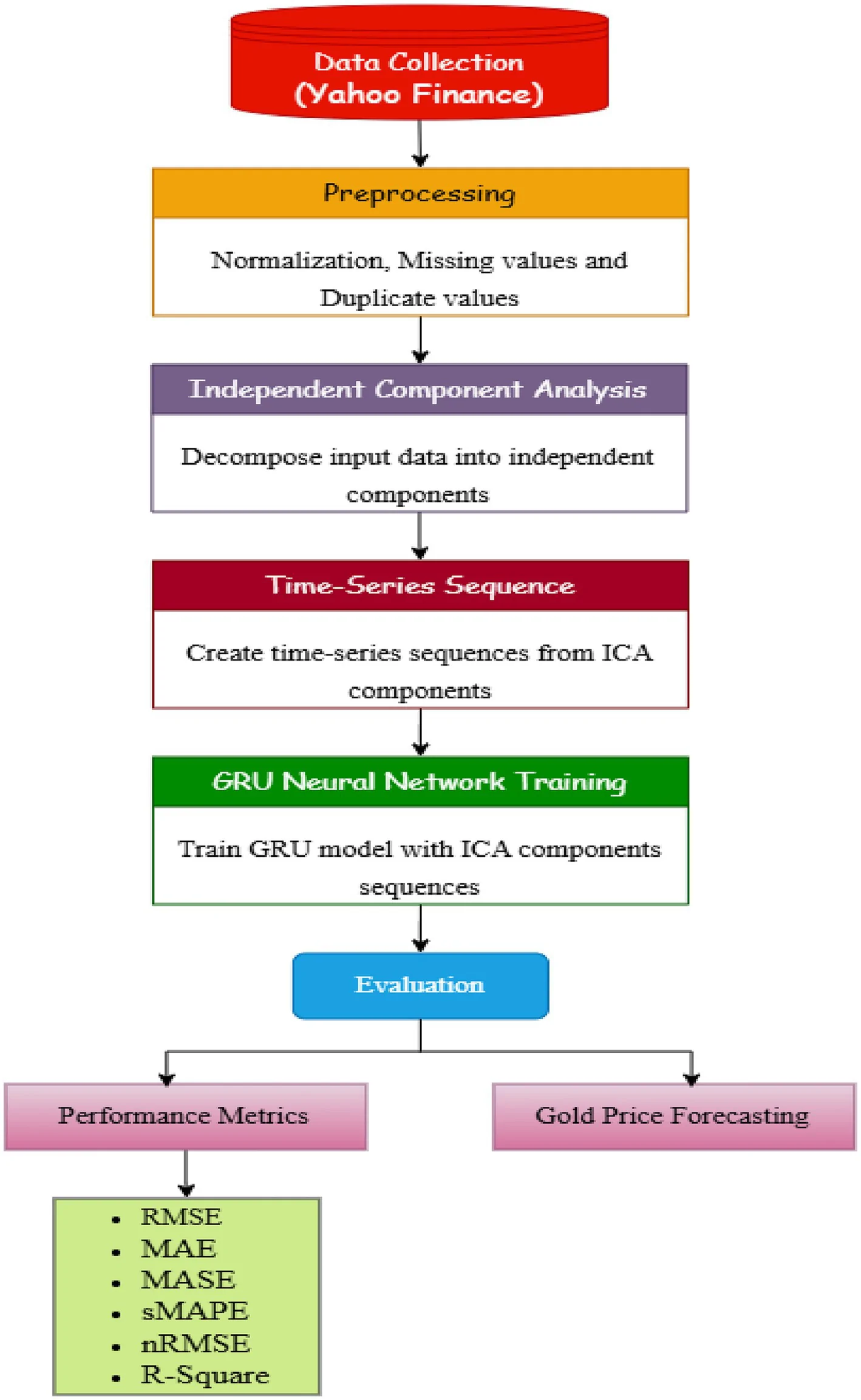
ICA-GRU network model for gold futures price forecasting.

The proposed forecasting framework integrates the Independent Component Analysis–Gated Recurrent Unit (ICA-GRU) network, as illustrated in Figure 2, to develop and evaluate a hybrid deep learning model for gold futures price forecasting. The framework combines statistical signal decomposition with deep learning techniques to improve forecasting accuracy by effectively capturing the nonlinear, volatile, and temporal characteristics of gold futures price movements.

#### 3.1.1 Dataset description

Table 1 presents the features of the dataset used for gold futures price forecasting. Historical daily gold futures data were obtained from Yahoo Finance using the ticker symbol GC=F, covering the period from 1 January 2015 to 29 January 2026. The dataset was downloaded on 3 February 2026 and consisted of 2,784 daily observations containing five market variables: Open, High, Low, Close, and Trading Volume. The daily Close price was selected as the target variable because the Yahoo Finance GC=F dataset reports the official daily closing (settlement) price for the continuous futures contract, while the remaining variables (Open, High, Low, and Volume) served as predictor variables for model development.

**Table 1:** Dataset description.

Attribute	Description
Data source	Yahoo finance
Asset	Gold futures (GC=F)
Data frequency	Daily
Study period	01-01-2015–01-29-2026
Variables	Open, High, Low, Close, Volume
Daily observations	2,784
Data type	Time-series data
Target variable	Closing price

The Yahoo Finance ticker symbol GC=F represents the continuous COMEX Gold Futures Contract rather than an individual expiring futures contract. Unlike single futures contracts that expire periodically, the continuous futures series is generated through the automatic rolling of expiring contracts into the next actively traded contract, thereby providing a continuous historical price series suitable for long-term forecasting analysis. The use of a continuous futures series avoids interruptions associated with contract expiration and enables the proposed deep learning model to learn long-term temporal dependencies from an uninterrupted sequence of observations. Although continuous futures contracts may experience minor price adjustments around contract roll dates, no additional manual roll adjustments were performed because the continuous GC=F series obtained from Yahoo Finance was used directly throughout the analysis.

#### 3.1.2 Data preprocessing

Before model training, a comprehensive preprocessing procedure was performed to improve data quality and learning efficiency. The dataset was examined for duplicate records and missing observations. A total of 5 missing observations were handled using the forward-fill imputation method, which preserves the continuity of financial time-series data by replacing missing values with the most recent valid observation (Usmani et al., 2024). All preprocessing parameters, including the z-score standardization statistics (mean and standard deviation), Min-Max normalization parameters, and the ICA transformation matrix, were estimated exclusively from the training dataset. The learned transformations were subsequently applied to the testing dataset without refitting, thereby preventing information leakage and ensuring that no information from the testing period influenced model development. Following missing-value treatment, the predictor variables (Open, High, Low, and Volume) were standardized using z-score normalization to ensure equal feature contribution and improve numerical stability during model training (Lima and Souza, 2023; Park et al., 2022). The standardization process is expressed in Equation (1):

$$x^{\prime }=\frac{x-\mu }{\sigma }$$ (1)

Where *x*′ = Standardized value of the input feature, *x* = Original value of the input feature (Open, High, Low or Volume), *μ* = Mean of the corresponding input feature and *σ* = Standard deviation of the corresponding input feature. This equation transforms each input feature to have a mean of zero and a standard deviation of one, ensuring that all features contribute equally during model training.

Subsequently, the target variable (Close Price) was normalized using the Min–Max scaling technique, as presented in Equation 2, to transform the values into the range of 0–1 and facilitate faster model convergence. The preprocessing framework enhances the reliability, stability, and predictive capability of the proposed ICA-GRU forecasting system.

$$y^{\prime }=\frac{y-y_{\min}}{y_{\max}-y_{\min}}$$ (2)

Where *y*′ = Normalized value of the target variable, *y* = Original close price, *y*_min_ = Minimum closing price in the dataset, *y*_max_ = Maximum closing price in the dataset.

To obtain statistically independent feature representations from correlated financial variables, Independent Component Analysis (ICA) was applied to the standardized input feature matrix. The ICA technique decomposes observed multivariate data into independent components by optimizing for non-Gaussianity (Shu et al., 2023). The FastICA algorithm was employed because of its computational efficiency and rapid convergence in estimating statistically independent components. Assume that (*X*) is the standardized input matrix; then ICA converts (*X*) as presented in Equation (3).

S=WX (3)

where (*S*) represents the matrix of independent components and (*W*) is the unmixing matrix. These components serve as refined inputs for the subsequent sequence modeling stage.

The dataset was arranged chronologically to preserve the temporal ordering of the gold-price series. After constructing 30-day input sequences, the first 80% of supervised observations were used as the development set and the final 20% were retained as an unseen test set. The final test set contained 557 observations. Within the development set, the final 10% was used for validation and hyperparameter selection. All feature scalers, target scalers, PCA transformations and FastICA transformations were fitted exclusively using the available development or training observations and were then applied unchanged to subsequent validation or test observations. No test observations were used during transformation fitting, model selection or epoch selection.

To represent temporal relations, the transformed data was converted into supervised learning sequences using a sliding window technique. A window size of 30 time steps was used; the observation window included the past 30 trading days, and the output value was the closing price the next day (Casolaro et al., 2023). Window sizes of 10, 30, and 60 trading days were evaluated under identical experimental conditions, and the 30-day window achieved the best overall forecasting performance. Therefore, it was adopted for all subsequent experiments. The proposed ICA-GRU model performs single-step-ahead forecasting. Specifically, each input sequence consists of the previous 30 consecutive trading days, and the model predicts only the closing price of the immediately following trading day (*t* + 1). Multi-step forecasting, in which prices are predicted for several future trading days simultaneously or recursively, was beyond the scope of the present study. Therefore, all reported forecasting results correspond to one-day-ahead predictions. Mathematically, for time step (*t*), the input–output pairs were formulated in Equation (4):

$$X_{t}=[S_{t-30},S_{t-29},\ldots ,S_{t-1}],y_{t}=\text{Clos}e_{t}$$ (4)

This formulation enables the model to learn both short-term and long-term temporal patterns in the data.

The neural forecasting models predicted the next-day closing-price change rather than the closing-price level directly. The final closing-price forecast was reconstructed in Equation (5):

$${\hat{P}}_{t}=P_{t-1}+\hat{△P_{t}}$$ (5)

where $P_{t-1}$ is the most recently observed closing price and $\hat{\Delta P_{t}}$ is the predicted next-day price change.

#### 3.1.3 ICA-GRU model

The forecasting algorithm was developed using a stacked GRU model, which effectively captures temporal relations while avoiding the vanishing gradient problem (Zhang et al., 2024; Thor and Postek, 2024). The components of the model include: a GRU layer with 128 units, a second GRU layer with 64 units, a Dropout layer (rate = 0.2) after each GRU layer, and finally a fully connected dense layer with 1 unit. The gating mechanism (reset and update gate) helps GRU units to modify their hidden states.

Table 2 presents the recurrent forecasting model consisting of two GRU layers containing 128 and 64 hidden units, respectively, followed by dropout layers with a dropout rate of 0.20 and a dense output layer with one neuron. Adam optimizer with a learning rate of 0.001 (Lee and Kim, 2023; Reyad et al., 2023) and the mean squared error loss function was adopted for optimization using a batch size of 32. During model selection, training was performed for a maximum of 200 epochs with early stopping (patience = 20), and the final number of training epochs was selected according to validation performance. Candidate ICA and PCA dimensions of 2, 3, 4 and 5 were evaluated. To improve the robustness of the experimental results, LSTM, Standard GRU, PCA-GRU and ICA-GRU were evaluated using five independent random seeds (11, 22, 33, 44 and 55), and the mean and standard deviation were reported for all neural-network performance measures. The output layer contains a single neuron because the proposed network is designed to generate a one-day-ahead forecast for each input sequence.

**Table 2:** Hyperparameter configuration of the proposed ICA-GRU model.

Hyperparameter	Value
ICA components	2
Sequence length	30 trading days
GRU layer 1 units	128
GRU layer 2 units	64
Dropout rate	0.20
Dense output layer	1 Neuron
Optimizer	Adam
Learning rate	0.001
Loss function	Mean squared error (MSE)
Batch size	32
Epochs	200
Validation split	10%
Train-test split	80:20
Early stopping	Applied

#### 3.1.4 Benchmark models

To evaluate the effectiveness of the proposed ICA-GRU framework, seven benchmark forecasting models were implemented under identical experimental conditions. These include Persistence Forecast, Random Walk with Drift, Linear Autoregressive Model (AR(30)), Linear Regression, Long Short-Term Memory (LSTM), Standard GRU, and Principal Component Analysis integrated with GRU (PCA-GRU). All neural-network models used the same chronological data split, sequence length, optimizer, batch size, hidden-layer configuration, dropout rate, and evaluation protocol to ensure a fair comparison.

1. Persistence Forecast

The persistence model assumes that the next-day closing price is equal to the most recently observed closing price. It is widely used as a naïve benchmark in financial time-series forecasting, it is presented in Equation (6).

$${\hat{P}}_{t}=P_{t-1}$$ (6)

where $P_{t-1}$ denotes the observed closing price at time t−1 , and ${\hat{P}}_{t}$ is the predicted closing price.

1. Random Walk with Drift

The Random Walk with Drift model extends the persistence forecast by incorporating the average daily price change estimated from the training dataset as expressed in Equation (7).

$${\hat{P}}_{t}=P_{t-1}+{\bar{\mathrm{\Delta P}}}_{\text{train}}$$ (7)

where ${\bar{\mathrm{\Delta P}}}_{\text{train}}$ represents the average daily price change calculated from the development dataset.

1. Linear Autoregressive Model (AR(30))

The Linear Autoregressive model predicts the next-day price change using the previous 30 daily price changes as expressed in Equation (8).

$$\Delta P_{t}=\beta_{0}+\sum_{i=1}^{30}\beta_{i}\Delta P_{t-i}+\varepsilon_{t}$$ (8)

where $\Delta P_{t}$ is the current price change, $\beta_{i}$ are the autoregressive coefficients, and $\varepsilon_{t}$ is the random error term.

1. Linear Regression

The Linear Regression model uses the same 30-day multivariate input window as the neural-network models and is presented in Equation (9). The predictor variables include Open, High, Low, Volume, and Previous Close.

$$\hat{y}=\beta_{0}+\sum_{j=1}^{p}\beta_{j}x_{j}+\varepsilon$$ (9)

where $x_{j}$ denotes the predictor variables, $\beta_{j}$ are regression coefficients, and ε is the error term.

1. Long Short-Term Memory (LSTM)

The LSTM model was included as a recurrent neural-network benchmark because of its ability to capture long-term temporal dependencies through gated memory cells. The hidden state is updated as presented in Equation (10).

$$h_{t}=o_{t}⊙\tanh(c_{t})$$ (10)

where $h_{t}$ denotes the hidden state, $c_{t}$ is the cell state, $o_{t}$ is the output gate, and ⊙ represents element-wise multiplication.

1. Standard GRU

The Standard GRU model employed the same network architecture and hyperparameters as the proposed ICA-GRU model but used the original standardized predictor variables without feature decomposition. The GRU hidden state is computed using the following Equation (11).

$$h_{t}=(1-z_{t})⊙h_{t-1}+z_{t}⊙{\tilde{h}}_{t}$$ (11)

where $z_{t}$ is the update gate, $h_{t-1}$ is the previous hidden state, and ${\tilde{h}}_{t}$ is the candidate hidden variance, as presented in Equation (12).

1. PCA-GRU

The PCA-GRU model applies Principal Component Analysis (PCA) before the GRU network to reduce feature dimensionality while retaining most of the data variance.

Z=XW (12)

where X is the standardized feature matrix, W is the projection matrix formed from the principal component eigenvectors, and Z represents the transformed feature matrix used as input to the GRU network.

To further evaluate the contribution of the proposed ICA-GRU framework, additional decomposition methods were implemented under identical experimental conditions. These included Singular Spectrum Analysis integrated with GRU (SSA-GRU), Variational Mode Decomposition integrated with GRU (VMD-GRU), and Ensemble Empirical Mode Decomposition integrated with GRU (EEMD-GRU). In all cases, the decomposition method was applied as a preprocessing step before the GRU network. The same sequence length, training-test split, target definition, optimizer, batch size, network architecture, dropout rate, and evaluation metrics were maintained across all models. This ensured that any differences in forecasting performance could be attributed to the decomposition technique rather than differences in model architecture or training configuration.

Similarly, benchmark forecasting models including Persistence Forecast, Random Walk with Drift, Linear Autoregressive Model (AR(30)), Linear Regression, Long Short-Term Memory (LSTM), Standard GRU, and PCA-GRU were evaluated using the same experimental protocol. These models represent statistical, machine learning, and deep learning forecasting approaches commonly employed in financial time-series analysis. Their inclusion enables a comprehensive assessment of the proposed ICA-GRU framework against both conventional forecasting techniques and recent deep learning models.

#### 3.1.5 Performance evaluation

The performance of the proposed ICA-GRU model was evaluated using both regression and classification metrics to assess prediction accuracy and directional forecast capability comprehensively. The regression performance was measured using Root Mean Square Error (RMSE), Mean Absolute Error (MAE), Mean Absolute Scaled Error (MASE), Symmetric Mean Absolute Percentage Error (sMAPE) normalized RMSE and the Coefficient of Determination (*R*^2^), which quantify prediction error, average deviation, and the goodness-of-fit between actual and predicted values (Liang et al., 2022). To evaluate the model’s ability to predict the direction of gold futures price movements, forecasted price changes were transformed into binary classes (upward or downward), and classification performance was assessed using Precision, Recall, and the Confusion Matrix (Zhang et al., 2024).

To determine whether the observed improvement in forecasting performance was statistically significant, the prediction errors of the proposed ICA-GRU and baseline GRU models were compared using two complementary statistical procedures. First, the Wilcoxon Signed-Rank Test was applied to the paired absolute prediction errors to evaluate whether the median prediction errors differed significantly between the two models without assuming normality. The analysis also reported the sample size, median loss difference, effect size, treatment of zero differences, and a 95% confidence interval for the median loss difference. Second, a Diebold–Mariano (DM) test with a Newey–West heteroskedasticity-and-autocorrelation-consistent (HAC) variance estimator was conducted to compare forecast accuracy while accounting for potential serial correlation in time-series prediction errors. A significance level of 5% (*α* = 0.05) was adopted for all statistical analyses.

## 4 Results and data analysis

### 4.1 Gold futures price trend analysis

**Figure 3:**
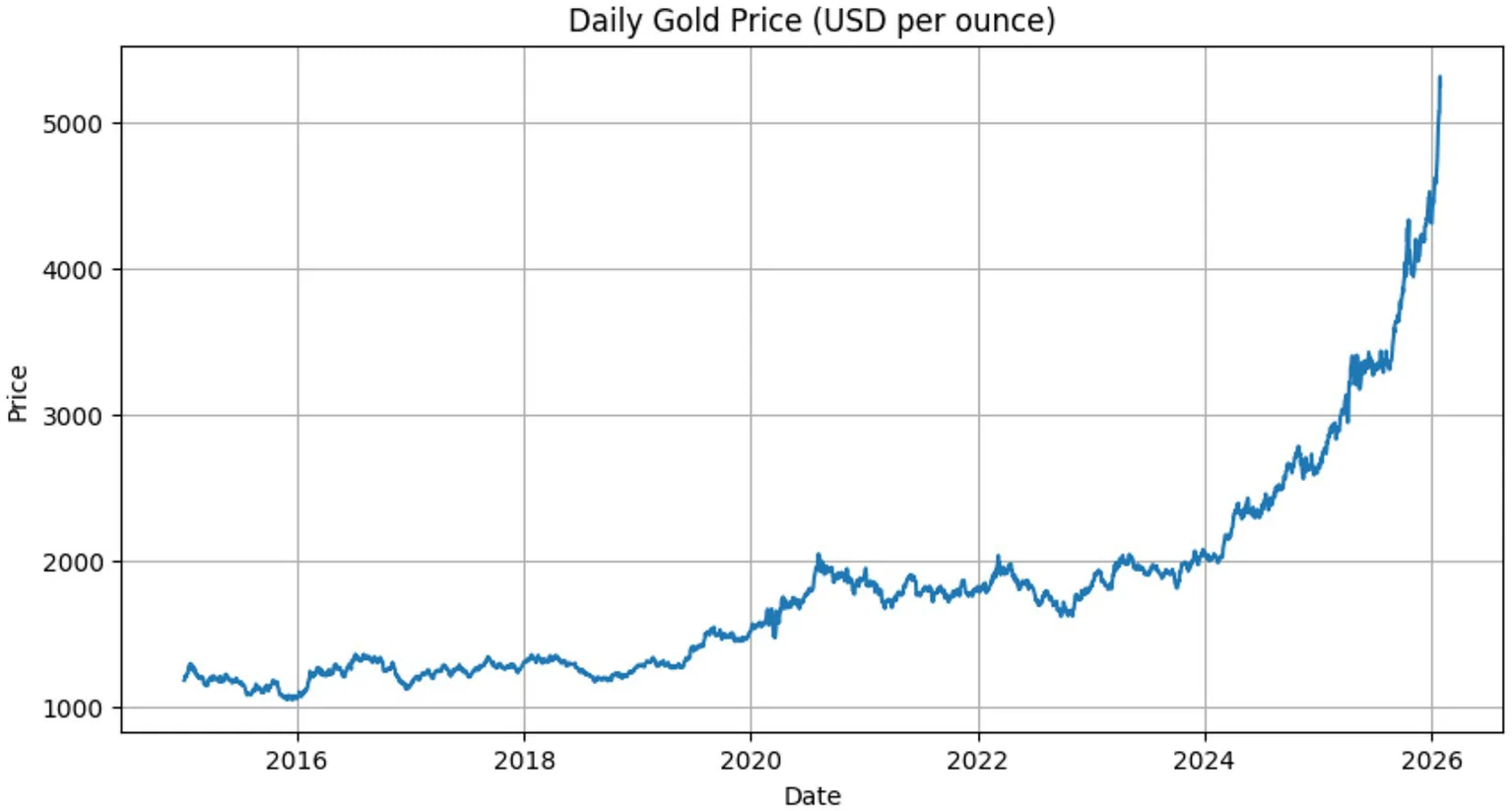
Gold futures price trend analysis.

Figure 3 illustrates the trend in daily gold futures prices in USD per ounce for the period from 2015 to 2026. Between 2015 and 2019, gold futures prices fluctuate slightly and, therefore, show low volatility. Between 2019 and 2021, there is a consistent growth in the price of gold, indicating the impact of increasing demand and uncertainty of the market. Between 2021 and 2023, gold futures prices fluctuate moderately, indicating the consolidation phase of the market with mixed signals. However, a dramatic change occurs after 2024 as gold futures prices experience tremendous increases in terms of high volatility. In 2025 and 2026, gold futures prices experience a further increase in volatility, reaching their peak in the entire period under consideration.

Gold displays a unique and clear performance pattern. Although the average return on gold is similar to that from stock investments, but the power of gold shows up in the short-run period, which lies between 1 and 3 years in which gold outperforms most other asset categories, including both stocks and bonds. This finding further demonstrates the effectiveness of gold as a protective asset during periods of financial uncertainty.

### 4.2 Forecasting performance of the proposed ICA-GRU model

Table 3 shows the forecasting performance of the suggested ICA-GRU model through the measures of Root Mean Square Error (RMSE), Mean Absolute Error (MAE), Mean Absolute Scaled Error (MASE) and coefficient of determination (*R*^2^). In this case, the proposed ICA-GRU model achieved RMSE of 39.36, MASE of 2.61 and MAE of 26.35, showing low forecasting errors and high prediction accuracy. The low values of the RMSE and MAE imply that the forecasted gold futures prices were close to the actual prices in the market. This shows that the suggested model was capable of forecasting the nonlinear and temporal relationships existing in gold futures price data. Additionally, the model produced an *R*^2^ value of 0.99, meaning that about 99% of the variation in gold futures prices was attributed to the proposed ICA-GRU model. Therefore, the results show the high explanatory and predictive capabilities of the suggested ICA-GRU model due to its ability to predict future values accurately.

**Table 3:** Performance metrics of model evaluation.

Metrics	Performance
RMSE	39.36
MAE	26.35
MASE	2.61
R-Square	0.99

### 4.3 Actual vs. predicted gold futures price analysis

**Figure 4:**
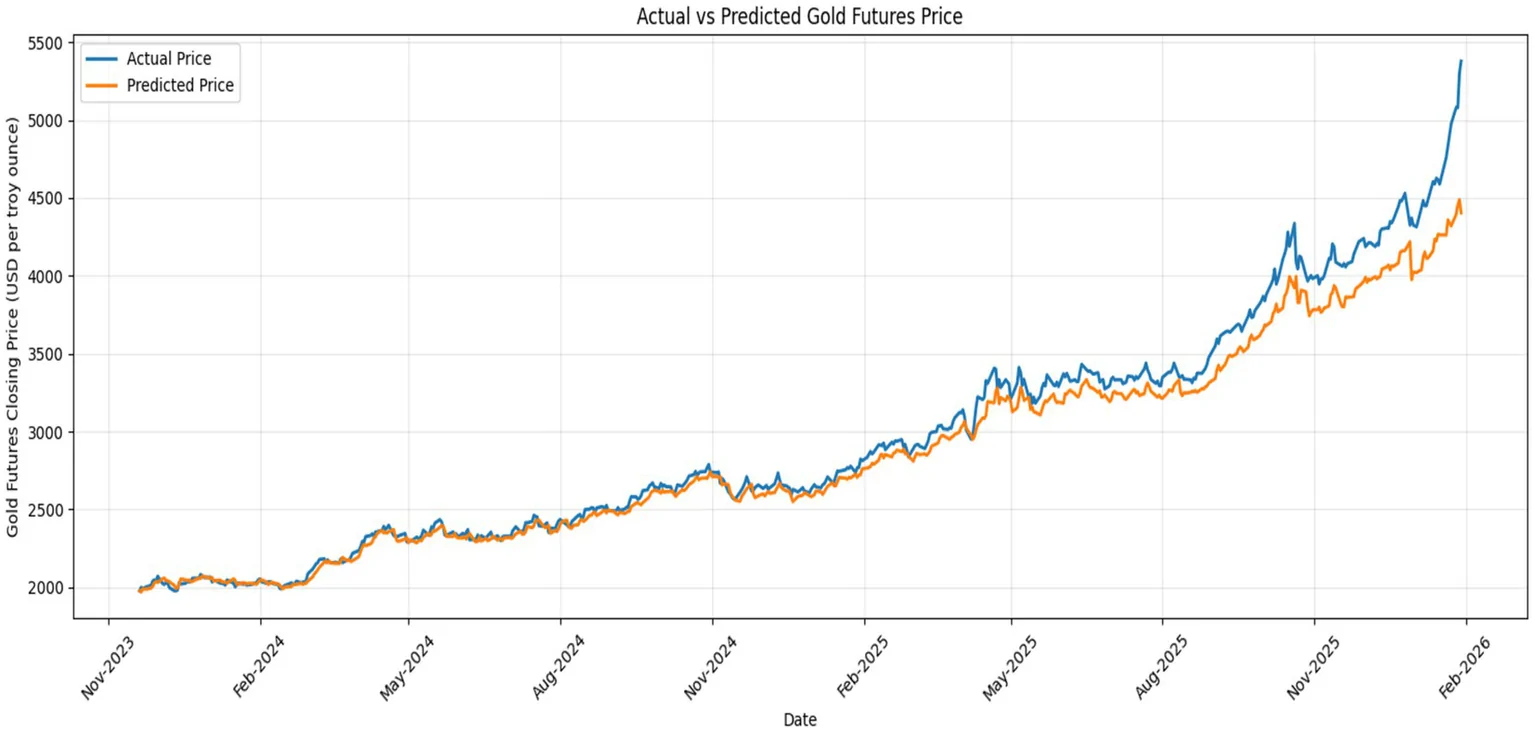
Actual vs. predicted gold futures price.

Figure 4 illustrates the actual and forecasted closing prices of gold from the early part of 2024 to the early part of 2026. From the actual price graph, it can be seen that there is a strong positive trend, which represents the substantial increase in gold futures prices during the period of the study. The forecasted prices follow the same trend, which indicates that the developed ICA-GRU model is effective in tracing the trend and behavior of gold futures prices. Although the forecasted prices are slightly smoother and lower than the actual prices, they still follow the trend and behavior of the actual prices effectively. The slight difference between the two graphs indicates mild understatement in forecasting prices when they are rapidly increasing, which is normal in financial time series that are highly volatile.

### 4.4 Error analysis

**Figure 5:**
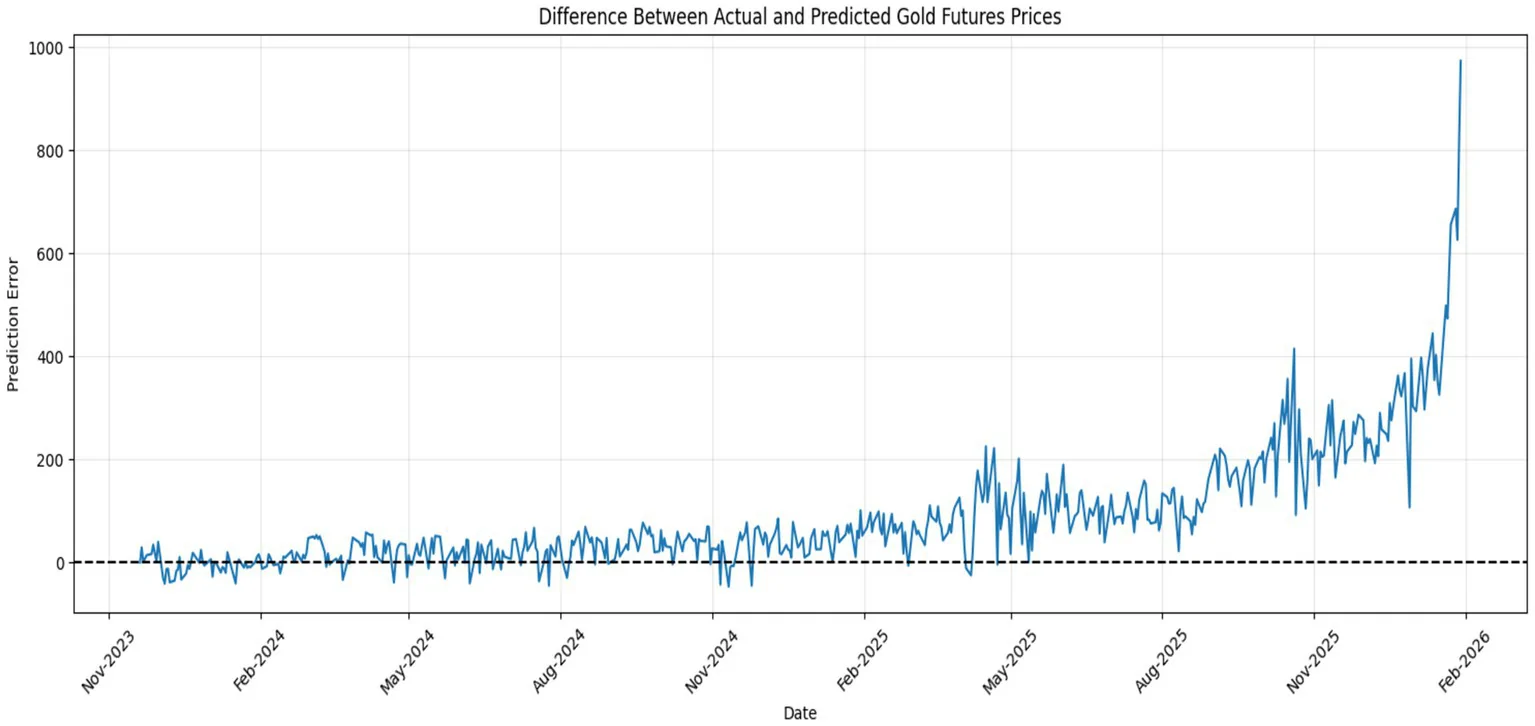
Difference between actual and predicted gold futures prices.

Figure 5 shows that prediction errors increase during the latter part of the testing period, particularly when gold futures prices experience rapid upward movements and heightened market volatility. Under these conditions, the proposed ICA-GRU model tends to underestimate extreme price increases, resulting in larger positive prediction errors. This behavior is common in financial time-series forecasting because sudden market movements are inherently difficult to capture using historical observations alone. Nevertheless, despite the increase in prediction errors during highly volatile periods, the proposed ICA-GRU model continues to follow the overall market trend and achieves strong overall forecasting performance (RMSE = 39.36, MAE = 26.35, and *R*^2^ = 0.99). Therefore, the model remains suitable for one-day-ahead forecasting under normal and moderately volatile market conditions, although additional exogenous variables or attention-based architectures may further improve performance during extreme market events.

### 4.5 Directional prediction performance

**Figure 6:**
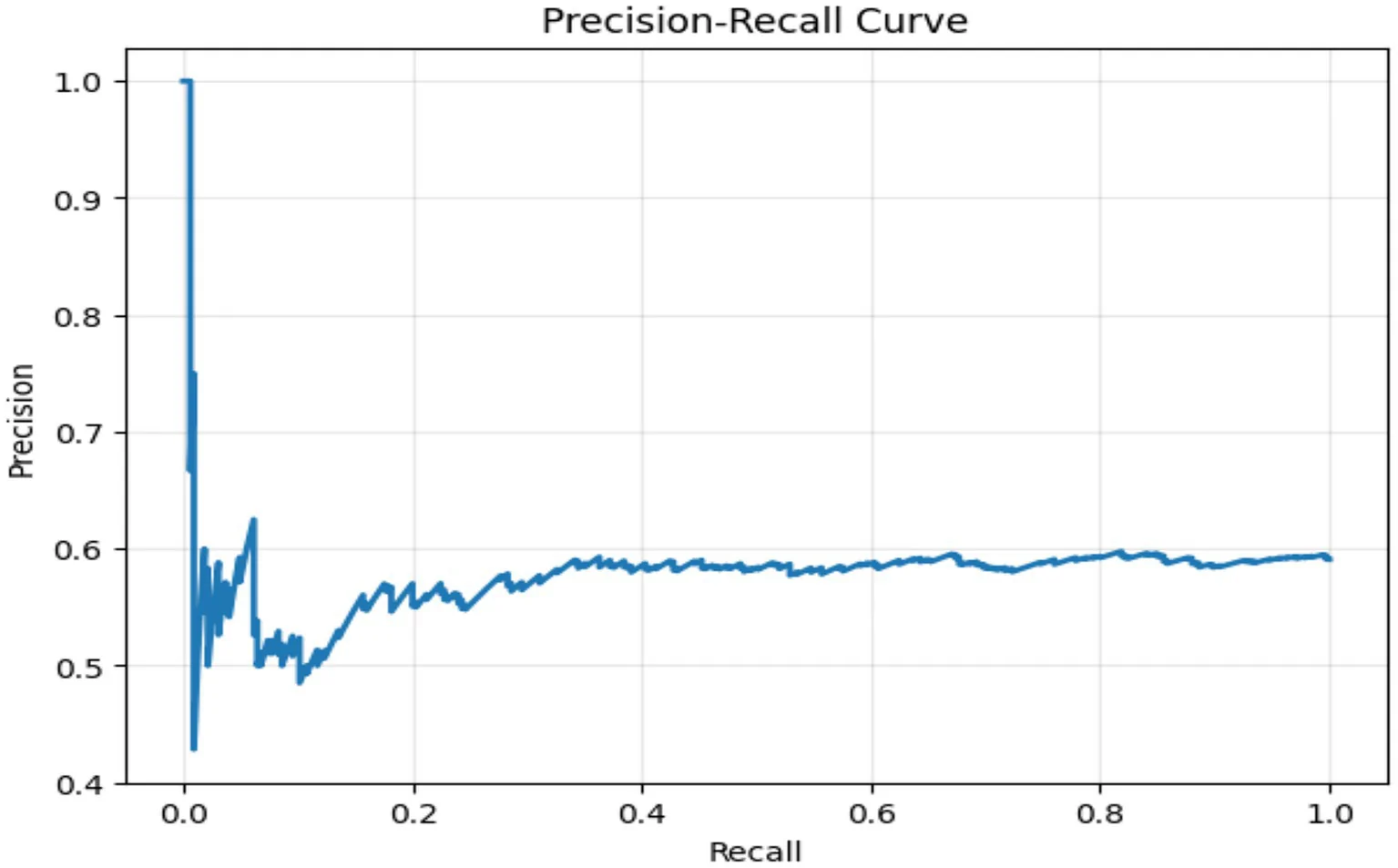
Precision and recall.

Figure 6 illustrates the Precision-Recall curve of the proposed ICA-GRU model for predicting the movement direction of gold futures prices. Precision refers to the accuracy of the predicted upward movements, while recall reflects the ability of the proposed ICA-GRU model to detect the true upward movements of the gold futures price. With very low recall values, the precision values are very high but show large variations, implying that with only a few predictions, the model shows great accuracy in predicting the upward price movement. With increasing recall values, the precision values first decrease because of the incorporation of less accurate predictions. The precision values are then stabilized and lie in the range of about 0.55–0.60 with an increase in recall values. This indicates that the proposed ICA-GRU model strikes a good balance between detecting true upward price movements and avoiding false positives.

**Figure 7:**
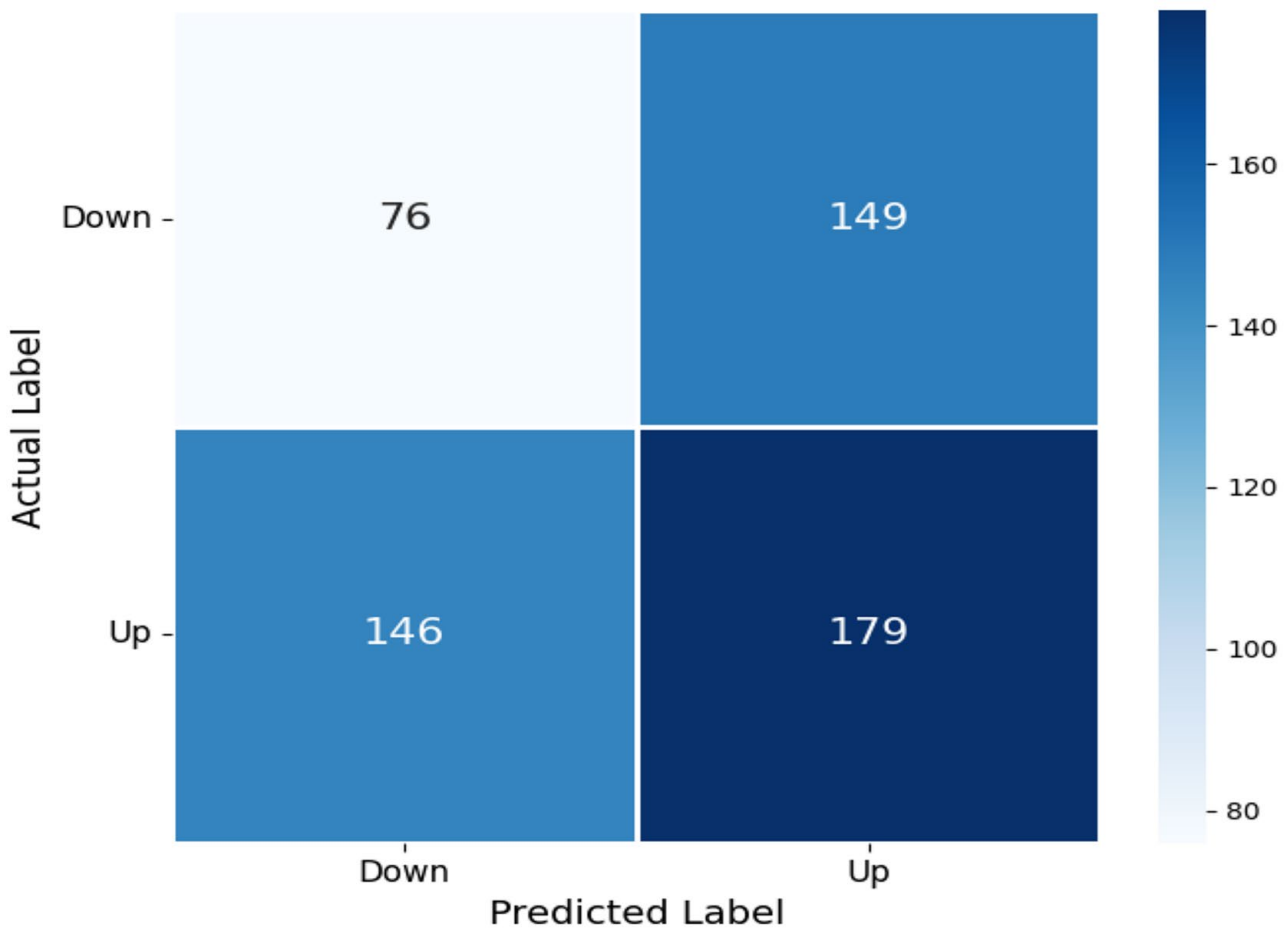
Confusion matrix.

Figure 7 presents the confusion matrix for the ICA-GRU model’s performance in predicting the direction of gold futures price movement (Up or Down). The rows in the confusion matrix correspond to actual gold futures price movements, while the columns correspond to the predicted movements. From the total number of observations, the model can correctly predict 76 downward movements and 179 upward movements. However, it incorrectly predicts 149 downward movements as upward movements and 146 upward movements as downward movements. The model has a stronger tendency to predict upward gold futures price movements, as evident from the fact that it has a relatively higher number of correct predictions for upward movements. The model’s bias in predicting upward gold futures price movements may be affected by the overall upward trend in gold futures prices that have been witnessed in the past few years. The model’s performance in predicting downward gold futures price movements is relatively weaker.

### 4.6 Comparative performance analysis

Table 4 compares the forecasting performance of the proposed ICA-GRU model with statistical, machine-learning, and deep-learning benchmark models. ICA-GRU achieved the lowest RMSE of 39.3699, MAE of 26.3552, MASE of 2.6179, sMAPE of 0.8546%, and nRMSE of 1.3410%, indicating the highest forecasting accuracy among all models. It also achieved the highest positive out-of-sample *R*^2^ of 0.0027, demonstrating better generalization to unseen data. Although all models reported similar *R*^2^ values between 0.9900 and 0.9921, ICA-GRU consistently produced lower prediction errors with *R*^2^ of 0.9971. These findings confirm that ICA-GRU provides more reliable and accurate forecasts than the benchmark forecasting models.

**Table 4:** Forecasting performance of ICA-GRU with benchmark models.

Model	RMSE	MAE	MASE	sMAPE (%)	Normalized RMSE (%)	$R^{2}$	Out-of-sample $R^{2}$
Persistence	39.4232	26.4165	2.6240	0.8567	1.3428	0.9921	0.0000
Random walk with drift	40.3901	27.2639	2.7082	0.8816	1.3757	0.9909	−0.0497
Linear AR	40.0282	27.1095	2.6928	0.8793	1.3634	0.9910	−0.0309
Linear regression	40.8128	27.9407	2.7754	0.9073	1.3901	0.9909	−0.0717
LSTM	39.5462 ± 0.1070	26.4847 ± 0.1026	2.6308 ± 0.0102	0.8580 ± 0.0032	1.3470 ± 0.0036	0.9911 ± 0.0000	−0.0063 ± 0.0054
Standard GRU	39.7700 ± 0.1942	26.6757 ± 0.1798	2.6497 ± 0.0179	0.8635 ± 0.0055	1.3546 ± 0.0066	0.9901 ± 0.0000	−0.0177 ± 0.0100
PCA-GRU	40.1194 ± 0.1079	27.0073 ± 0.1041	2.6827 ± 0.0103	0.8739 ± 0.0033	1.3665 ± 0.0037	0.9900 ± 0.0000	−0.0356 ± 0.0056
**ICA-GRU**	**39.3699 ± 0.1593**	**26.3552 ± 0.1418**	**2.6179 ± 0.0141**	**0.8546 ± 0.0043**	**1.3410 ± 0.0054**	**0.9971 ± 0.0000**	**0.0027 ± 0.0083**

The values in the bold are represents proposed ICA-GRU model indices.

### 4.7 Comparison of alternative decomposition methods

Table 5 compares the forecasting performance of different decomposition methods combined with the GRU model. Among the evaluated approaches, SSA-GRU achieved the lowest RMSE 39.7481, MAE 26.6559, MASE 2.6937, sMAPE 0.8629%, and NRMSE 1.3538%, indicating slightly better forecasting accuracy than the other decomposition-based models. VMD-GRU achieved performance comparable to SSA-GRU, whereas ICA-GRU, PCA-GRU, and EEMD-GRU showed relatively higher prediction errors. All decomposition-based models produced very similar *R*^2^ values between 0.9970 and 0.9971, indicating that the choice of decomposition method had only a marginal influence on forecasting performance under the common GRU backbone.

**Table 5:** Comparison of alternative decomposition methods under the common GRU backbone.

Model	RMSE	MAE	MASE	sMAPE (%)	NRMSE (%)	$R^{2}$	OOS $R^{2}$
ICA-GRU	40.3142 ± 0.1644	27.1951 ± 0.1389	2.7481 ± 0.0140	0.8796 ± 0.0042	1.3731 ± 0.0056	0.9970 ± 0.0000	−0.0457 ± 0.0085
SSA-GRU	39.7481 ± 0.1991	26.6559 ± 0.1843	2.6937 ± 0.0186	0.8629 ± 0.0057	1.3538 ± 0.0068	0.9971 ± 0.0000	−0.0166 ± 0.0102
VMD-GRU	39.7530 ± 0.2036	26.6593 ± 0.1882	2.6940 ± 0.0190	0.8630 ± 0.0058	1.3540 ± 0.0069	0.9971 ± 0.0000	−0.0168 ± 0.0104
EEMD-GRU	39.8119 ± 0.1188	26.7178 ± 0.1026	2.6999 ± 0.0104	0.8648 ± 0.0030	1.3560 ± 0.0040	0.9971 ± 0.0000	−0.0198 ± 0.0061

### 4.8 Computational cost and complexity analysis

Table 6 summarizes the experimental configuration used to evaluate the proposed forecasting model. The experiments were conducted on the Google Colab platform using a Linux operating system with Python 3.12.13 and TensorFlow 2.20.0. The dataset comprised 2,227 training sequences and 557 test sequences, with a sequence length of 30 and five input variables. The selected ICA components were used as inputs to the GRU model.

**Table 6:** Hardware and software environment.

Item	Configuration
Platform	Google Colab
Operating system	Linux 6.6.122+, x86-64
CPU model	Intel® Xeon® CPU @ 2.20 GHz
Physical CPU cores	1
Logical CPU cores	2
System RAM	12.67 GB
GPU	No GPU detected
Python	3.12.13
TensorFlow	2.20.0
Training sequences	2,227
Test sequences	557
Sequence length	30
Input variables	5
Selected ICA components	2

Table 7 presents the computational performance of the proposed ICA-GRU model. The model selected two ICA components and contained 88,001 trainable parameters. ICA preprocessing required only 0.0461 s, while the average training and test inference times were 45.7430 and 0.6755 s, respectively. The model achieved an inference latency of 1.2260 ms per sample. These results demonstrate that the proposed model is computationally efficient and suitable for practical forecasting applications without requiring GPU acceleration.

**Table 7:** Computational cost of the proposed ICA-GRU model.

Measure	Result
ICA components	2
Trainable parameters	88,001
ICA preprocessing time	0.0461 s
Mean training time	45.7430 ± 2.8453 s
Mean test-set inference time	0.6755 ± 0.0856 s
Mean inference latency	1.2260 ms/sample
Test observations	557
Approximate recurrent MACs per forecast	2,603,520
Selected training epochs	200
Processing device	CPU

### 4.9 Statistical significance analysis

#### 4.9.1 Wilcoxon signed-rank test

To determine whether the observed improvement in forecasting performance was statistically significant, the paired complete prediction errors of the proposed ICA-GRU model and the baseline GRU model were compared using the Wilcoxon Signed-Rank Test. Since the corresponding prediction errors did not require the assumption of normality, the Wilcoxon test was considered appropriate for evaluating differences in forecasting accuracy. The analysis included 557 paired observations with no zero-difference pairs.

As presented in Table 8, the Wilcoxon Signed-Rank Test produced a statistic of 143,008 with *p* < 0.001, indicating a statistically significant difference between the forecasting performances of the two models. The proposed ICA-GRU framework reduced the median absolute prediction error from 82.97 to 24.09, corresponding to a median improvement of 57.49. Furthermore, the large effect size (*r* = 0.763) and the 95% confidence interval that excludes zero provide strong evidence that the observed improvement is both statistically and practically significant.

**Table 8:** Statistical significance comparison between GRU and ICA-GRU models.

Measure	Value
Number of paired observations (*n*)	557
Zero differences	0
Wilcoxon signed-rank statistic (W)	143,008
*p*-value	<0.001
Median absolute error (GRU)	82.97
Median absolute error (ICA-GRU)	24.09
Median reduction in absolute prediction error (GRU − ICA-GRU)	57.49
Effect size (*r*)	0.763 (Large)
95% Confidence interval of median loss difference	[51.39, 63.56]

#### 4.9.2 Diebold–Mariano test

To further evaluate predictive performance under time-series dependence, the Diebold–Mariano (DM) test was applied using the absolute prediction errors of the GRU and ICA-GRU models. The analysis employed a Newey–West heteroskedasticity-and-autocorrelation-consistent (HAC) variance estimator to account for potential serial correlation in the forecast errors.

The results presented in Table 9 indicate that the proposed ICA-GRU model achieved significantly higher forecasting accuracy than the baseline GRU model. The positive mean loss difference confirms that the GRU model produced larger prediction errors, while the DM statistic (10.246, *p* < 0.001) demonstrates that this improvement is statistically significant even after accounting for serial correlation in the forecast errors. These findings further support the robustness of the proposed ICA-GRU forecasting framework.

**Table 9:** Diebold–Mariano test results for comparing the forecasting accuracy of the GRU and ICA-GRU models.

Measure	Value
Number of observations (*n*)	557
Loss function	Absolute prediction error
Mean loss difference (GRU − ICA-GRU)	62.65
HAC variance estimator	Newey–West
HAC lag length	8
Diebold–Mariano statistic	10.246
*p*-value	<0.001

### 4.10 Comparison with existing studies

Table 10 provides a qualitative comparison between the proposed ICA-GRU framework and representative gold futures price forecasting approaches reported in the recent literature. Previous studies have employed machine learning models with technical indicators, CNN-LSTM architectures, encoder-decoder networks, BiLSTM models, and image-based deep learning frameworks to capture nonlinear market dynamics (Priambodo et al., 2025; Mohtasham Khani et al., 2021; Salim and Djunaidy, 2024). In contrast, the proposed ICA-GRU model integrates Independent Component Analysis (ICA) with a stacked GRU architecture to generate statistically independent feature representations before temporal sequence learning. Since the reviewed studies differ in datasets, forecasting horizons, preprocessing strategies, and evaluation protocols, the comparison highlights methodological differences rather than direct numerical superiority.

**Table 10:** Comparison of representative gold futures price forecasting approaches.

Study	Data source	Forecasting model	Main contribution
(Priambodo et al., 2025)	Yahoo finance	MLR, SVM and CNN-LSTM = Fibonacci retracement	Combined technical indicators with machine learning models to improve forecasting.
(Mohtasham Khani et al., 2021)	Yahoo finance	CNN-LSTM, Encoder-Decoder-LSTM, BiLSTM	Investigated deep learning architectures for gold futures price forecasting during pandemic conditions.
(Salim and Djunaidy, 2024)	Yahoo finance	CNN-LSTM with Gramian Angular Field (GAF)	Converted time-series data into image representations for CNN-based forecasting.
Present Study	Yahoo finance	ICA-GRU network	Integrates Independent Component Analysis for feature decomposition with stacked GRU for temporal learning.

## 5 Discussion

The results from the empirical analysis demonstrate that the suggested ICA-GRU model offers high prediction accuracy regarding the prices of gold and can successfully capture the temporal dynamics of this asset. The evaluation results show that the suggested model has achieved an RMSE of 39.36, an MAE of 26.35, MASE of 2.61 and *R*^2^ equal to 0.99. Given the low forecast errors, one may assume that the predicted prices of gold have approximated the real prices on the market, while the extremely high value of the coefficient of determination proves that approximately 99% of the variation in gold futures prices was captured by the suggested model.

Analysis of real price and forecasted price also highlights the efficiency of the suggested framework. The forecasted price trend matches the real price movement of gold almost accurately. Even though the forecasted price trend shows less volatility compared to the actual price trend during price spikes, the degree of similarity in both price trends is still significant. This smoothing behavior is one of the usual features of deep learning forecast models that try to reduce the forecast error while retaining their generalization ability.

Moreover, the analysis of errors confirms once again the validity of the proposed ICA-GRU model. The lack of any tendency to error accumulation testifies to the stability of the model’s forecasting ability. Despite some periods of increased forecasting errors in times of high market volatility, they all stay within admissible limits and do not influence the general forecasting ability of the model. The predominance of positive forecast errors confirms the slightly underestimating nature of the model’s forecast in the case of fast price rises.

From the directional prediction analysis, it is clear that the suggested ICA-GRU model is capable of predicting the future movement of gold futures price directions moderately, yet meaningfully. From the Precision-Recall analysis, it becomes evident that the ratio between precision and recall scores is relatively stable; that is, precision scores stay mostly in the range of 0.55–0.60, irrespective of various recall scores. Thus, we can say that the model is better at predicting directions of gold futures price movements in comparison with random classification. Additionally, from the results of the confusion matrix analysis, it becomes apparent that the model is better at predicting upward movements of gold futures prices compared to the downward ones. Such a tendency might be because there was an upward trend in gold futures prices during the research period.

The comparative analysis demonstrates that the proposed ICA-GRU model achieved better forecasting performance than the standard GRU model under the same experimental conditions. The ICA-GRU model obtained lower forecasting errors RMSE = 39.37, MAE = 26.36, and MASE = 2.62 than the standard GRU model RMSE = 39.77, MAE = 26.68, and MASE = 2.65, while both models achieved comparable *R*^2^ values. Furthermore, the ICA-GRU model produced a positive out-of-sample *R*^2^ of 0.0027, whereas the standard GRU model reported a negative value of −0.0177, indicating improved generalization to unseen observations. The Wilcoxon Signed-Rank Test *p* < 0.001 and the Diebold–Mariano test *p* < 0.001 further confirmed that the observed improvement was statistically significant. These findings suggest that incorporating Independent Component Analysis before GRU learning can improve forecasting performance compared with the conventional GRU model.

The comparison with previous studies provides a methodological perspective. Earlier studies adopted diverse forecasting frameworks, including CNN-LSTM, BiLSTM, encoder-decoder architectures and technical indicator-based machine learning models. However, these studies differ in datasets, study periods, forecasting horizons, preprocessing techniques, feature sets and evaluation protocols, making direct numerical comparisons inappropriate. Therefore, the findings of the present study primarily demonstrate the effectiveness of the proposed ICA-GRU model within the experimental setting. Furthermore, the decomposition analysis indicates that reducing feature dependence alone does not necessarily improve forecasting performance. Although ICA successfully transformed the input variables into statistically independent components, PCA achieved a similar reduction in correlation without improving predictive accuracy. Among the decomposition-based models, SSA-GRU and VMD-GRU produced slightly better results than the standard GRU, while ICA-GRU and PCA-GRU showed comparatively lower performance. Although SSA-GRU achieved slightly lower forecasting errors than ICA-GRU, the performance differences were marginal. These findings indicate that ICA remains an effective feature decomposition technique, while the suitability of a decomposition method depends on the characteristics of the underlying financial time-series dataset. These results suggest that preserving temporal characteristics through decomposition methods such as SSA and VMD is more beneficial than applying global latent transformations for the present dataset. Overall, the results demonstrate that increasing model complexity through ICA preprocessing does not necessarily lead to improved out-of-sample forecasting accuracy.

Although SSA-GRU achieved marginally lower forecasting errors than ICA-GRU under the present experimental setting, the primary objective of this study was not to identify the single best decomposition method, but rather to investigate the effectiveness of ICA as a computationally efficient feature transformation technique integrated with a GRU network for gold futures price forecasting. The comparison with alternative decomposition methods was conducted as a robustness analysis to evaluate the relative performance of ICA against other representative decomposition approaches. Therefore, the proposed ICA-GRU framework remains the principal contribution of this study, while the competitive performance of SSA-GRU highlights an important direction for future research on adaptive and hybrid decomposition strategies.

In summary, the empirical findings indicate that the presented ICA-GRU model is a reliable, effective, and statistically proven solution for forecasting gold futures prices. The proposed ICA-GRU model demonstrated competitive forecasting performance and outperformed several benchmark forecasting models, while the comparative decomposition analysis indicated that the effectiveness of feature decomposition techniques depends on the characteristics of the underlying dataset. The combination of feature decomposition approaches, deep temporal models, and regularization methods results in higher prediction accuracy and model stability; thus, the presented framework can be considered as an efficient tool for financial forecasting purposes.

## 6 Limitations and future scope

From the application point of view, the model would work best in making predictions about the levels of prices at both short- and long-term horizons and performing trend analysis in markets where most of the activity is based on trends. However, while forecasts regarding the direction of prices are somewhat reliable, they do not appear to be very reliable in the case of declines, and this might be an issue for models sensitive to risks. Besides, the performance of the model was assessed on a specific data set with specific features (OHLC, volume). Therefore, the proposed ICA-GRU model does not explicitly account for external information sources such as investor sentiment, macroeconomic conditions, or market uncertainty indicators, which may influence gold futures price movements, particularly during periods of heightened market volatility. Furthermore, although the proposed ICA-GRU framework achieved competitive forecasting performance, the decomposition analysis showed that SSA-GRU produced marginally lower forecasting errors under the current experimental setting. This suggests that the effectiveness of feature decomposition techniques may depend on the characteristics of the underlying dataset and forecasting task.

Future research activities would involve current research framework as follows: (i) Training models with knowledge about the regimes (either separate models or a classifier of regimes to feed into the GRU network), (ii) Applying asymmetric loss function to better reflect the under-prediction of the drawdowns, (iii) Using exogenous factors, including investor sentiment indices, macroeconomic variables such as USD Index, interest rates, inflation proxies, and market uncertainty indicators like volatility indices, (iv) Investigating hybrid decomposition approaches that combine ICA with methods such as SSA or VMD, or developing adaptive decomposition strategies that automatically select the most suitable decomposition technique according to changing market conditions, and (iv) Estimation of uncertainty (prediction intervals).

## 7 Conclusion

The suggested framework is a hybrid ICA-GRU model that uses the signal decomposition technique of ICA along with the stacked architecture of GRU. The findings prove that this proposed ICA-GRU model is capable of modeling nonlinear relations in gold futures price time series data and provides better prediction results than several other forecasting methods available today. It also emphasizes the significance of feature decomposition, sequence construction, and regularization techniques in improving forecast accuracy.

Three key findings from the experiment include: First, that statistically based pre-processing methods, such as ICA, are highly effective at boosting the accuracy of deep learning models used for financial forecasting. Second, that recurrent models, like the GRU network, perform exceptionally well for forecasting gold futures prices. Third, the integration of feature decomposition techniques with deep recurrent architectures significantly improves forecasting performance and model robustness. Thus, the proposed ICA-GRU system is an innovative, scalable forecasting approach that could be very useful in predicting gold futures prices.

## Data Availability

The raw data supporting the conclusions of this article will be made available by the authors, without undue reservation.
